# Investigating the Association of Pain Intensity and Health Status among Older US Adults with Pain Who Used Opioids in 2020 Using the Medical Expenditure Panel Survey

**DOI:** 10.3390/healthcare11142010

**Published:** 2023-07-12

**Authors:** David R. Axon, Taylor Maldonado

**Affiliations:** 1 Department of Pharmacy Practice & Science, R. Ken Coit College of Pharmacy, The University of Arizona, 1295 N. Martin Ave., Tucson, AZ 85721, USA; tamaldonado0812@arizona.edu; 2 Center for Health Outcomes and PharmacoEconomic Research (HOPE Center), R. Ken Coit College of Pharmacy, The University of Arizona, 1295 N. Martin Ave., Tucson, AZ 85721, USA

**Keywords:** pain, health status, opioids, older adults

## Abstract

The number of older United States (US) adults is increasing, yet extra life years are not always spent in good health. This study explored the relationship between pain intensity and health status among US adults aged ≥50 with pain who used an opioid in the 2020 Medical Expenditure Panel Survey using multivariable logistic regression adjusting for demographic, economic, and health variables. Most (60.2%) older US adult opioid users with pain reported having good health (versus 39.8% poor health). In the fully adjusted analysis, those with extreme pain (odds ratio (OR) = 0.19, 95% confidence interval (CI) = 0.10, 0.35) and quite a bit of pain (OR = 0.34, 95% CI = 0.19, 0.60) had lower odds of reporting good health compared to those with little pain. There was no statistical relationship between health status for moderate versus little pain. In addition, males (versus females; OR = 0.61, 95% CI = 0.40, 0.91), white race (versus not white; OR = 0.43, 95% CI = 0.22, 0.84), education ≤high school (versus >high school; OR = 0.61, 95% CI = 0.41, 0.92), and current smoker (versus non-smoker; OR = 0.55, 95% CI = 0.32, 0.93) were associated with lower odds of reporting good health. Being employed (versus unemployed; OR = 1.88, 95% CI = 1.06, 3.33), having <2 chronic conditions (versus ≥2; OR = 4.38, 95% CI = 1.91, 10.02), and doing regular physical activity (versus not; OR = 2.69, 95% CI = 1.73, 4.19) were associated with higher odds of reporting good health. These variables should be considered when assessing the health needs and developing treatment plans for older US adult opioid users with pain.

## 1. Introduction

As of 2022, adults aged 50 and older make up over one-third (36%) of the United States (US) population, which is projected to increase over the next few decades [1,2]. Although people are living longer, they are not necessarily living healthier, which presents a public health challenge that needs to be addressed [3]. As adults age, they have an increased risk for diseases such as dementia, heart disease, arthritis, and cancer [2].

Pain is defined as “an unpleasant sensory and emotional experience associated with, or resembling that associated with, actual or potential tissue damage” by the International Association for the Study of Pain [4]. While 50.2 million US adults experience pain every day, approximately 100 million US adults have reported experiencing some amount of chronic pain [5]. A study using the 2011 National Health and Aging Trends Study showed that 52.9% of people over the age of 65 years old reported had bothersome pain in the last month. Those who were experiencing pain had a 72% higher rate of reporting an inability to walk three blocks, compared to those not experiencing pain, which hinders good quality of life [6].

Appropriate pain management for older adults is a difficult clinical task for many physicians [7]. Opioids are considered the most potent pain relievers and are often a patient’s best option for pain relief despite their highly addictive nature [7,8]. Millions of Americans are treated with opioids annually despite their addictive potential and known side effects including nausea, vomiting, and constipation [8,9]. In a study using the 2015 Medical Expenditure Panel Survey (MEPS), 32.9% of older adults who experienced pain in the past four weeks reported using an opioid [10]. More recent data suggest 22.1% of US adults had an opioid prescription for chronic pain in 2019. Of these, the 45–64-year-old age group constituted approximately 26% of prescriptions, but people aged over 65 years old saw a decrease in opioid usage compared to the 45–64 years age group [11]. The average cost of a single person living with an opioid use disorder is about $221,219 a year (measured in 2017 US dollars), however, this estimate varies across the US. The opioid epidemic is a serious public health issue that led to 47,000 US adult deaths in 2018 [12]. In addition, opioid misuse among Medicare beneficiaries was associated with substance abuse, psychiatric disease, and pain [13]. There is therefore an interest and a need for further interest in opioid users in the US.

Seventy million US adults over the age of 50 have at least one chronic illness [14]. Chronic pain affects more than 50% of US older adults [7] and is rarely ever the sole symptom in older age. Pain is often paired with fatigue, mood disturbance, sleep disturbances, and mobility disorders [15]. A study performed on patients with high-intensity lower back pain found they had a decrease in general well-being compared to those who do not experience intense pain [16].

Previous research shows that 73.4% of older US adults experiencing pain reported good health and of US older adults (age ≥ 50 years) 8.9% reported excellent health, 28.3% reported very good health, 36.2% reported good health, and 26.6% reported fair/poor health [17,18]. While these studies analyzed the association between health status and self-reported pain, the existing body of literature pertaining to the specific context of pain and opioid use among older US adults is deemed insufficient. Given the substantial impact of opioid use in the US, research is warranted to investigate the association of pain severity and health status among older US adults who used opioids, to identify any specific differences between this targeted population and other populations. This study, therefore, sought to assess the association between pain intensity and health status among older US adults with pain who used opioids. This information may be useful to better understand the factors associated with health status among older US adults with pain who use opioids and raise awareness of the prevalence of opioid use among this population. This information may be useful for targeted public health interventions and in clinical decision-making.

## 2. Materials and Methods

### 2.1. Medical Expenditure Panel Survey (MEPS)

For this study, we merged the 2020 Medical Expenditure Panel Survey (MEPS) full-year consolidated data file with the 2020 MEPS prescribed medicines file. These datasets combined provided data on many demographic, economic, and health variables for 27,805 civilian, non-institutionalized citizens in the US. Using the sampling framework from the previous year’s National Health Interview Survey, the MEPS staff surveyed eligible participants multiple times over a two-year period to collect data. These data are then supplemented by data provided by insurance providers and medical providers [19,20,21].

### 2.2. Inclusion Criteria and Study Subjects

We included individuals from the 2020 MEPS data in our study if they were alive throughout 2020, were at least 50 years of age, had pain in the past four weeks, and used an opioid medication in 2020. We defined an individual with pain as someone who responded extremely, quite a bit, moderately, or a little bit when asked: “During the past four weeks, how much did pain interfere with your normal work (including both work outside the home and housework)?” in the full-year consolidated data file [19,20,21]. We defined opioid use using the Multum Lexicon therapeutic class codes for narcotic analgesics or combination narcotic analgesics in the 2020 prescribed medicines file [19,20,21].

### 2.3. Variables

Our independent variable was pain intensity, developed from the same MEPS item described above. We used four levels for this variable: extreme pain, quite a bit of pain, moderate pain, and little pain.

Our dependent variable was health status. We categorized health status as good or poor based on participants’ responded to items that asked how they would describe their health in general. We categorized responses of excellent, very good, and good as good health, and responses of fair or poor health as poor health.

We also included relevant demographic, economic, and health variables in our study. Demographic variables included status on age, sex, ethnicity, and race. Economic variables included education, employment, income, and marriage. Health variables included being overweight, chronic conditions, regular physical activity, and current smoker status [19,20,21].

### 2.4. Study Design and Analysis

We used a cross-sectional, retrospective study design. We compared the differences between the good and poor health groups using a chi-squared test. We performed an unadjusted logistic regression analysis to assess the association between levels of pain severity (our independent variable) and health status (our dependent variable). Then, we performed three multivariable logistic regression analyses. In the first multivariable analysis, we included pain severity and adjusted for demographic variables. In the second multivariable analysis, we included pain severity and adjusted for demographic and economic variables. In the third multivariable analysis, we included pain severity and adjusted for demographic, economic, and health variables. In all regression analyses, poor health served as the reference group. Statistical significance for odds ratios was determined based on the confidence intervals, where a confidence interval that included 1 indicated no statistical significance between the comparisons. We used the appropriate weighting variable to obtain nationally representative estimates and maintained the clusters and strata within the complex survey data. We calculated standard errors using the Taylor-series linearization approach. We set an a priori alpha level of 0.05. We used SAS on demand for academics (SAS Institute Inc., Cary, NC, USA) for all analyses.

## 3. Results

We included 844 individuals in the study, which represented a weighted population of 10,602,045 US older adults with pain who used an opioid in 2020. This was stratified as 500 people representing a weighted population of 6,384,326 (60.2%; 95% confidence interval (CI) = 55.8–64.6%) 2020 with good health and 344 people representing a weighted population of 4,217,719 (39.8%; 95% CI = 35.4–44.2%) with poor health.

We summarized the characteristics of the study participants in Table 1. The proportions of older US adults with pain who used opioids were similar among those with quite a bit (28.5%), moderate (27.0%), and little pain (27.4%), with 17.1% reporting extreme pain. Most individuals were ≥65 years old, female, non-Hispanic, white, had higher than high school education, were unemployed, had mid-high income, were married, were overweight, had ≥2 chronic conditions, did not regularly exercise, and were nonsmokers. We observed significant differences between groups for all variables except age, sex, marriage, and overweight status.

We summarized the results of the logistic regression analyses in Table 2. In the unadjusted analysis, those with extreme pain and quite a bit of pain had lower odds of reporting good health compared to those with little pain. There was no association between health status for moderate versus little pain. This remained the case in multivariable models. In the final multivariable model, males (versus females), white race (versus not white race), education ≤high school (versus >high school), and current smoker (versus not a current smoker) were associated with lower odds of reporting good health. Meanwhile, being employed (versus unemployed), having <2 chronic conditions (versus ≥2), and doing regular physical activity (versus not doing so) were associated with higher odds of reporting good health. All models had Likelihood Ratio tests and Wald tests <0.0001.

## 4. Discussion

This study describes the association between pain intensity and good health in US older adults with pain who used opioids in 2020. We also looked at demographic, economic, and health variables and their association with good health in US older adults with pain who used opioids. These findings are discussed in the context of existing knowledge below.

We found that, overall, 17.1% of US adults with pain who used opioids experienced extreme pain, 28.5% experienced quite a bit of pain, 27.0% experienced moderate pain, and 27.4% experienced little pain. Given that opioids are typically not a first-line therapy for pain management and are only suggested for use when the benefit outweighs the risk [22], it was interesting that over 50% of US older adults using opioids in this study only had little or moderate pain. Opioids are usually more appropriate for use among people who are experiencing moderate to extreme pain [23]. However, this finding may be due to the therapeutic effect of opioids reducing the pain intensity for these people, who consequently ranked their pain as little or moderate. Further investigation may be necessary to determine if opioids are being appropriately used in this population, or whether alternative therapies may be more appropriate. Many other pain management strategies exist that may adequately control pain without the side effects of opioids [24,25].

In the multivariate analysis, we observed those with extreme pain and those with quite a bit of pain were associated with lower odds of reporting good health when compared to those with little pain. However, we did not observe the same association among those with moderate pain. People with moderate pain report having a better quality of life which may result in better health status than those experiencing extreme pain and quite a bit of pain [26]. While there is more research on how the intensity of pain affects the quality of life, there is little literature associating pain intensity with good health. Our study offers some explanation of how older adults using opioids have their health affected by pain intensity. This could be helpful for clinicians developing care plans to ensure their patients are implementing healthy lifestyles to hopefully improve their health status.

Aside from pain intensity, we also found other variables associated with good health among US adults with pain who used opioids. The demographic variables that were associated with good health were sex and race. We found males had lower odds of reporting good health than females. Differences in health between sexes can be difficult to quantify and may be influenced by biological factors and cultural norms [27]. Biologically, it is thought that because the Y chromosome holds fewer genes than the X chromosome, males may lack a protective gene that would prevent them from developing disease. From a cultural perspective, males are sometimes more likely to engage in riskier health behaviors such as aggression, smoking, substance abuse, unhealthy diets, and refusing medical care compared to females [28], which may explain why males have lower odds of reporting good health in our study. Life expectancy offers an alternative perspective of health differences between males and females. Males had an average life expectancy of 73.2 years and females had an average life expectancy of 79.1 in 2021, which may be due to poorer health among males [29]. Our study findings lend additional evidence of differences between males and females in health status, particularly among this specific population of older US adults with pain who used opioids.

We also found those who identified as white had lower odds of reporting good health than those who did not identify as white. This finding contradicts what we might expect based on knowledge from the current literature. For instance, men who identify as African American are twice as likely to die from prostate cancer compared to men who identify as White, which may be due to the differences in healthcare received, a difference in genetic mutations, or a mixture of both [30]. One example of genetic differences between races is the mutation frequencies of the EGFR (epidermal growth factor receptor) gene (the typical target in lung cancer therapy) where mutations occur in Asian patients 30% of the time and only 7% in White patients [31]. However, there is a recognized implicit racial bias in healthcare that favors those identifying as White and helps improve their outcomes compared to members of other races in almost all instances [32]. Additional education (in professional training programs and as continuing education) for healthcare professionals about sex and race differences in health status is warranted to raise awareness and help address these issues.

Of the economic variables that were analyzed, we observed those with a high school education or less had lower odds of reporting good health than those who have more than a high school education, while those who were employed had higher odds of reporting good health than those who are unemployed. A recent meta-analysis corrected for publication bias showed that education had no positive effect on overall health in adults globally [33]. However, another review found that the level of education acquired is often recognized as a social determinant of health [34]. Having a higher level of education can help someone secure a better job that is more likely to offer health benefits, which increases access to healthcare services and ultimately improves health. People with further education are also more likely to understand their health needs and advocate for themselves [35]. To decrease this discrepancy in the future, perhaps further policies are needed that focus on establishing the foundations of a healthy lifestyle for students in the primary education system. A study using the 2002–2014 General Society Survey found that people working at higher quality jobs that paid more and presented less occupational risk reported better general health overall [36]. In addition, people with steady employment are more likely to be healthy than those who are unable to keep a job [37]. This information suggests a need for outreach to those without a job to provide any necessary healthcare services and help improve health outcomes.

Among the health variables analyzed, we observed having fewer chronic conditions and doing regular physical activity were associated with greater odds of reporting good health, while being a current smoker was associated with lower odds of reporting good health. These are unsurprising findings that are supported by existing knowledge but provide confirmatory evidence in this specific population of older US adults with pain who used opioids. Approximately 133 million Americans have at least one chronic disease, with hypertension being the most common [38]. Over 95% of older adults aged 65 years or older have at least one chronic disease and 80% have two or more [39]. While chronic illnesses often have specific symptoms associated with them, they can also bring about pain, mood disorders, and fatigue which all reduce health status [40]. A study conducted in Singapore found that individuals with more chronic diseases also had more issues with their overall well-being. Chronic diseases present issues with mobility, pain, limitations, and mental well-being [40]. Another study that conducted a literature review analyzing 70 studies found that in all cases of chronic disease, including heart disease, renal failure, diabetes, human immunodeficiency viruses, different cancers, and multiple sclerosis, patients had reduced health-related quality of life, which is important in determining health status [41]. Disease management and the physical toll on the patient’s body lead to less active and less motivated individuals, and more stress, which all lead to poorer health outcomes [42]. This knowledge supports our finding that US adults aged ≥50 years old who had pain and used opioids had over four times the odds of reporting good health if they had fewer chronic conditions (i.e., <2 chronic conditions) compared to those with multiple chronic conditions (i.e., ≥2 chronic conditions). Healthy behaviors like eating healthily, exercising regularly, not smoking, and practicing preventative medicine can decrease a person’s risk of developing a chronic disease [38]. Knowing that it is sometimes possible to prevent the development of chronic disease by implementing lifestyle changes, we recommend more education for the public about healthy lifestyles.

A study using 2020 MEPS data showed that people aged 60–69 years old with pain who used opioids had higher odds of reporting frequent physical exercise than people over 80 years old with pain who used opioids. The same study also suggested that the people reporting frequent physical exercise had better-perceived health than those who did not frequently exercise [43]. Given that physical activity has proven health benefits, such as improving brain health, managing weight, reducing the risk of disease, and strengthening bones and muscles [44,45], healthcare providers should consider the association of frequent physical exercise and health when creating care plans for patients with pain.

A study looking at the outcomes of lung cancer found 82% of people that are diagnosed with non-small cell lung cancer had been a smoker at some point in their life, and 41% were smokers at the time of diagnosis [46]. It is also known that the risk of smoking-related cardiovascular disease is affected by the number of pack-years that person has smoked [47]. Along with heart disease and lung cancer, smoking can also increase the risk of stroke, diabetes, and chronic obstructive pulmonary disease, which all contribute to the decline of a person’s health status [48]. The negative health effects found associated with smoking should influence policy enforcing stricter rules on cigarette and tobacco purchases, including e-cigarettes.

This study is vulnerable to some limitations, mainly due to the study design being unable to demonstrate a causal relationship. Participants may have overestimated or underestimated their true health status in this self-reported survey. Recall bias may also be present as respondents were asked to respond to the MEPS items over time. Finally, many of the variables in this analysis were dichotomous which may have reduced the granularity of the data.

## 5. Conclusions

The study was a cross-section of 844 US older adults with pain who used an opioid in 2020, representing approximately 10,602,045 US older adults with pain who used an opioid in 2020. We found that those who experienced extreme pain and quite a bit of pain (compared to little pain) were associated with lower odds of reporting good health. We also found that different demographic, economic, and health variables were associated with good health, including sex, race, degree of education, employment, chronic conditions, regular physical activity, and smoking status. This information can be used by healthcare providers when assessing the well-being and creating care plans for US older adults who used an opioid.

## Figures and Tables

**Table 1:** Characteristics of US adults with pain and opioid use in the study population.

		Good Health N = 500 % [95% CI]	Poor Health N = 344 % [95% CI]	Total N = 844 % [95% CI]	*p*
Pain intensity:					
Pain	Extreme	8.3 [5.3, 11.3]	30.5 [24.6, 36.3]	17.1 [14.0, 20.2]	<0.0001
	Quite a bit	22.4 [18.3, 26.6]	37.8 [31.7, 43.9]	28.5 [25.2, 31.8]	
	Moderate	33.1 [27.7, 38.5]	17.7 [12.7, 22.8]	27.0 [23.0, 30.9]	
	Little	36.2 [30.9, 41.5]	14.1 [10.0, 18.1]	27.4 [23.8, 31.0]	
Demographic variables:					
Age	50–64 years	43.5 [37.6, 49.3]	52.6 [45.4, 59.9]	47.1 [42.6, 51.6]	0.0535
	≥65 years	56.5 [50.7, 62.4]	47.4 [40.1, 54.6]	52.9 [48.4, 57.4]	
Sex	Male	37.0 [32.2, 41.9]	43.1 [36.3, 49.9]	39.4 [35.5, 43.4]	0.1525
	Female	63.0 [58.1, 67.8]	56.9 [50.1, 63.7]	60.6 [56.6, 64.5]	
Hispanic	Yes	4.1 [2.3, 6.0]	9.1 [5.2, 12.9]	6.1 [4.1, 8.2]	0.0042
	No	95.9 [94.0, 97.7]	90.9 [87.1, 94.8]	93.9 [91.8, 95.9]	
White race	Yes	85.9 [82.4, 89.5]	76.7 [71.4, 82.0]	82.3 [79.2, 85.3]	0.0026
	No	14.1 [10.5, 17.6]	23.3 [18.0, 28.6]	17.7 [14.7, 20.8]	
Economic variables:					
Education	≤High school	40.6 [36.3, 44.9]	59.9 [52.8, 67.0]	48.3 [44.4, 52.2]	<0.0001
	>High school	59.4 [55.1, 63.7]	40.1 [33.0, 47.2]	51.7 [47.8, 55.6]	
Employment	Employed	36.5 [31.2, 41.8]	15.7 [10.9, 20.4]	28.2 [24.3, 32.1]	<0.0001
	Unemployed	63.5 [58.2, 68.8]	84.3 [79.6, 89.1]	71.8 [67.9, 75.7]	
Income	Low	31.4 [26.4, 36.4]	46.1 [39.4, 52.7]	37.2 [33.0, 41.5]	0.0003
	Mid-high	68.6 [63.6, 73.6]	53.9 [47.3, 60.6]	62.8 [58.5, 67.0]	
Marriage	Married	51.0 [45.7, 56.3]	48.9 [42.2, 55.6]	50.1 [46.2, 54.1]	0.6462
	Not married	49.0 [43.7, 54.3]	51.1 [44.4, 57.8]	49.9 [45.9, 53.8]	
Health variables:					
Overweight	Yes	74.7 [69.5, 79.8]	78.0 [72.2, 83.8]	76.0 [72.0, 79.9]	0.3933
	No	25.3 [20.2, 30.5]	22.0 [16.2, 27.8]	24.0 [20.1, 28.0]	
Chronic conditions	<2	15.4 [11.6, 19.2]	4.3 [1.6, 6.9]	11.0 [8.3, 13.6]	<0.0001
	≥2	84.6 [80.8, 88.4]	95.7 [93.1, 98.4]	89.0 [86.4, 91.7]	
Regular physical activity	Yes	45.8 [40.8, 50.8]	20.3 [14.9, 25.8]	35.7 [31.6, 39.9]	<0.0001
	No	54.2 [49.2, 59.2]	79.7 [74.2, 85.1]	64.3 [60.1, 68.4]	
Current smoker	Yes	10.1 [7.1, 13.1]	23.1 [18.5, 27.8]	15.3 [12.6, 17.9]	<0.0001
	No	89.9 [86.9, 92.9]	76.9 [72.2, 81.5]	84.7 [82.1, 87.4]	

CI = confidence interval.

**Table 2:** Relationship between pain intensity and good health among US adults with pain and opioid use.

	Unadjusted Model	Adjusted for Demographic Variables	Adjusted for Demographic & Economic Variables	Adjusted for Demographic, Economic, & Health Variables
	Odds Ratio (95% CI)	Odds Ratio (95% CI)	Odds Ratio (95% CI)	Odds Ratio (95% CI)
Pain intensity:				
Extreme vs. little	**0.11 [0.06, 0.19]**	**0.11 [0.06, 0.20]**	**0.15 [0.08, 0.27]**	**0.19 [0.10, 0.35]**
Quite a bit vs. little	**0.23 [0.14, 0.38]**	**0.24 [0.14, 0.39]**	**0.30 [0.18, 0.51]**	**0.34 [0.19, 0.60]**
Moderate vs. little	0.73 [0.43, 1.22]	0.72 [0.42, 1.21]	0.75 [0.45, 1.26]	0.79 [0.46, 1.36]
Demographic variables:				
Age, 50–64 vs. ≥65 years		0.82 [0.56, 1.20]	0.72 [0.47, 1.10]	0.73 [0.48, 1.11]
Sex, male vs. female		0.71 [0.49, 1.02]	**0.67 [0.46, 0.98]**	**0.61 [0.40, 0.91]**
White race, yes vs. no		**0.45 [0.24, 0.84]**	**0.49 [0.25, 0.94]**	**0.43 [0.22, 0.84]**
Hispanic, yes vs. no		1.48 [0.96, 2.27]	1.44 [0.93, 2.24]	1.24 [0.77, 1.99]
Economic variables:				
Education, ≤high school vs. >high school			**0.59 [0.40, 0.88]**	**0.61 [0.41, 0.92]**
Employment, employed vs. unemployed			**2.30 [1.34, 3.94]**	**1.88 [1.06, 3.33]**
Income, low vs. mid-high			1.06 [0.68, 1.67]	1.04 [0.67, 1.63]
Marriage, married vs. not married			0.92 [0.60, 1.39]	0.76 [0.50, 1.17]
Health variables:				
Overweight, yes vs. no				0.94 [0.58, 1.53]
Chronic conditions, <2 vs. ≥2				**4.38 [1.91, 10.02]**
Regular physical activity, yes vs. no				**2.69 [1.73, 4.19]**
Current smoker, yes vs. no				**0.55 [0.32, 0.93]**

CI = confidence interval. Significant results are reported in **bold**.

## Data Availability

Data are available from the corresponding author upon reasonable request.
